# A case of paraprotein-negative POEMS syndrome: Case report and literature review

**DOI:** 10.1097/MD.0000000000039267

**Published:** 2024-09-06

**Authors:** Chao Ding, Yanqiu Li

**Affiliations:** Department of Hematology, Suining Central Hospital, Suining, China

**Keywords:** Castleman disease, lenalidomide, paraprotein-negative, POEMS syndrome

## Abstract

**Rationale:**

POEMS syndrome is a rare monoclonal plasma cell disease. The diagnosis of POEMS requires polyradiculoneuropathy and monoclonal plasma proliferating as 2 mandatory criteria, at least 1 of the major criteria (Castleman disease, elevated vascular endothelial growth factor level, and sclerotic bone lesion), and at least 1 of the minor criteria (organomegaly, extravascular volume overload, endocrinopathy, skin changes, papilledema, and thrombocytosis/polycythemia). This multisystem disorder is of high heterogeneity, and few variants of POEMS with no evidence of monoclonal gammopathy have been described, which further complicates the diagnosis in clinical practice. Now, we report a case of paraprotein-negative POEMS syndrome.

**Patients concerns:**

A 45-year-old woman complained of lower extremity edema, shortness of breath, abdominal distension, and lymphadenopathy for few years. Finally, she was diagnosed with paraprotein-negative POEMS syndrome. With the lenalidomide-based regimen, the symptoms were all relieved.

**Diagnosis:**

Paraprotein-negative POEMS syndrome.

**Intervention:**

Lenalidomide-based regimen and some supportive therapy.

**Outcome:**

All symptoms were relieved after 1 year of treatment.

**Lessons:**

Physicians should pay more attention to the POEMS syndrome, especially the POEMS syndrome variants, which are absence of paraprotein; probably, these variants are just “on the way” to classic POEMS syndrome antiplasma cell therapy, which remains the treatment of choice.

## 1. Introduction

POEMS syndrome is a rare monoclonal plasma cell disease involving multiple systems and is characterized by polyneuropathy, organomegaly, endocrinopathy, monoclonal plasma cell-proliferative disorder, and skin changes.^[1]^ POEMS syndrome is easy to misdiagnose and delays the diagnosis, especially M protein-negative POEMS syndrome due to the absence of clonal plasma cells. Herein, we report a case of paraprotein-negative POEMS syndrome.

## 2. Case information

**Figure 1.**
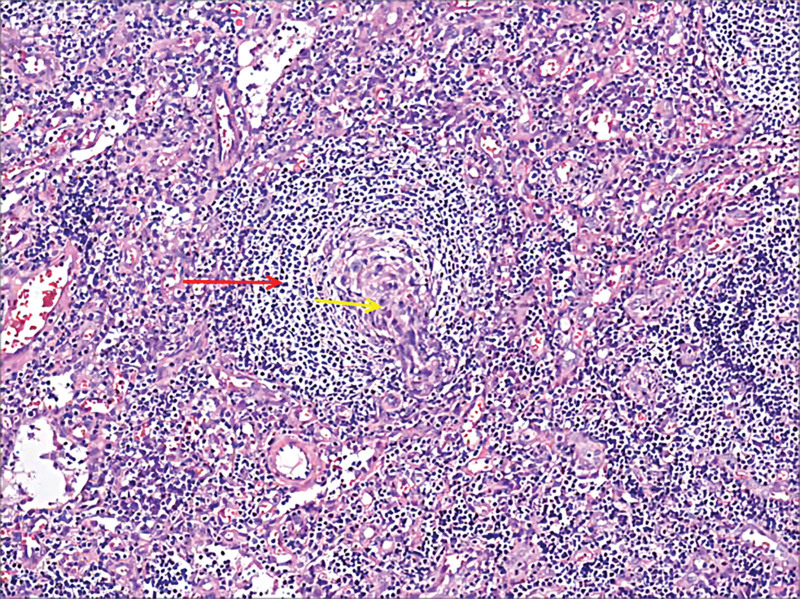
Pathological biopsy of the left axillary lymph node with atrophy of the follicular growth center with small blood vessels growing into it and hyperplastic sleeve cell areas arranged in concentric circles with an “onion skin”–like appearance (red arrows). “Onion-skin appearance” of the mantle zone around the germinal centers. The follicles were surrounded by prominent mantle zones containing small lymphocytes arranged in a concentric fashion (red arrow). The follicles were surrounded by prominent mantle zones containing small lymphocytes arranged in a concentric fashion (red arrow). “Lollipop appearance”: sclerotic blood vessels radially penetrated atrophic germinal centers (yellow arrow; magnification: 40×).

**Figure 2.**
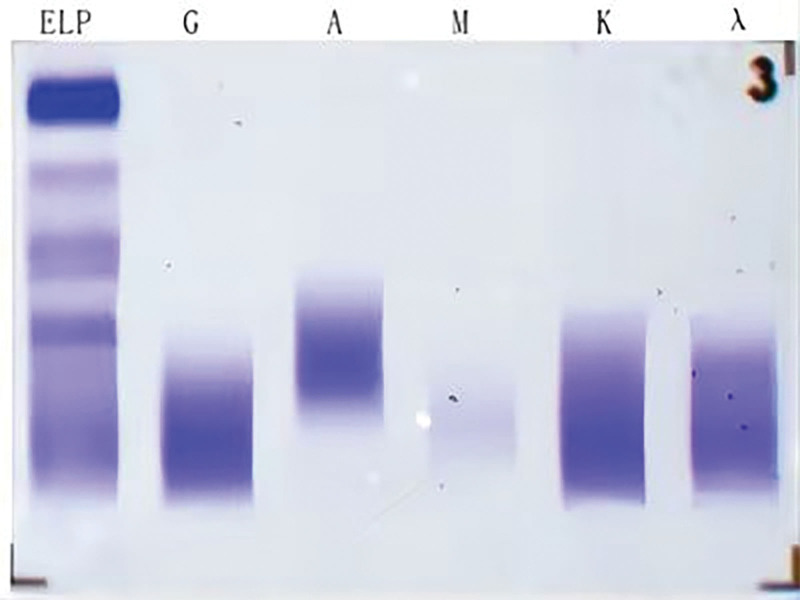
Serum immunofixation electrophoresis evidenced the absence of any detectable monoclonal immunoglobulin.

**Figure 3.**
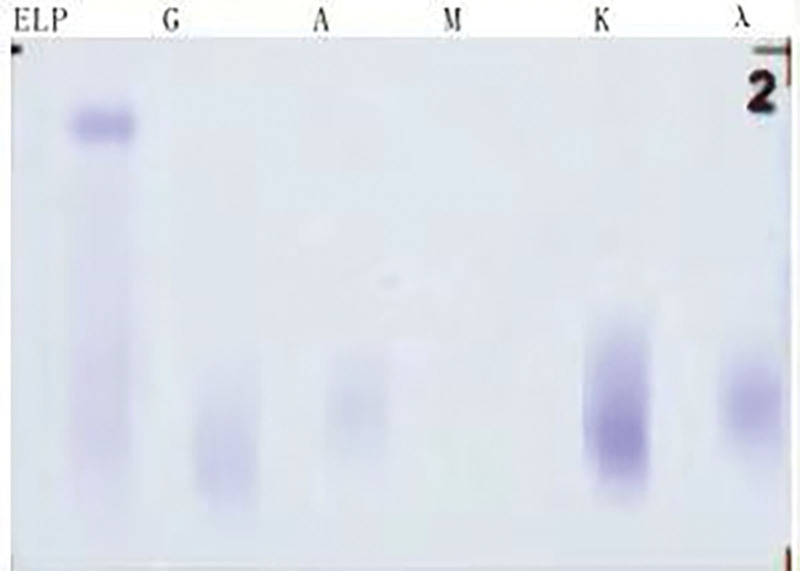
Urine immunofixation electrophoresis revealed the absence of any detectable monoclonal immunoglobulin.

**Figure 4.**
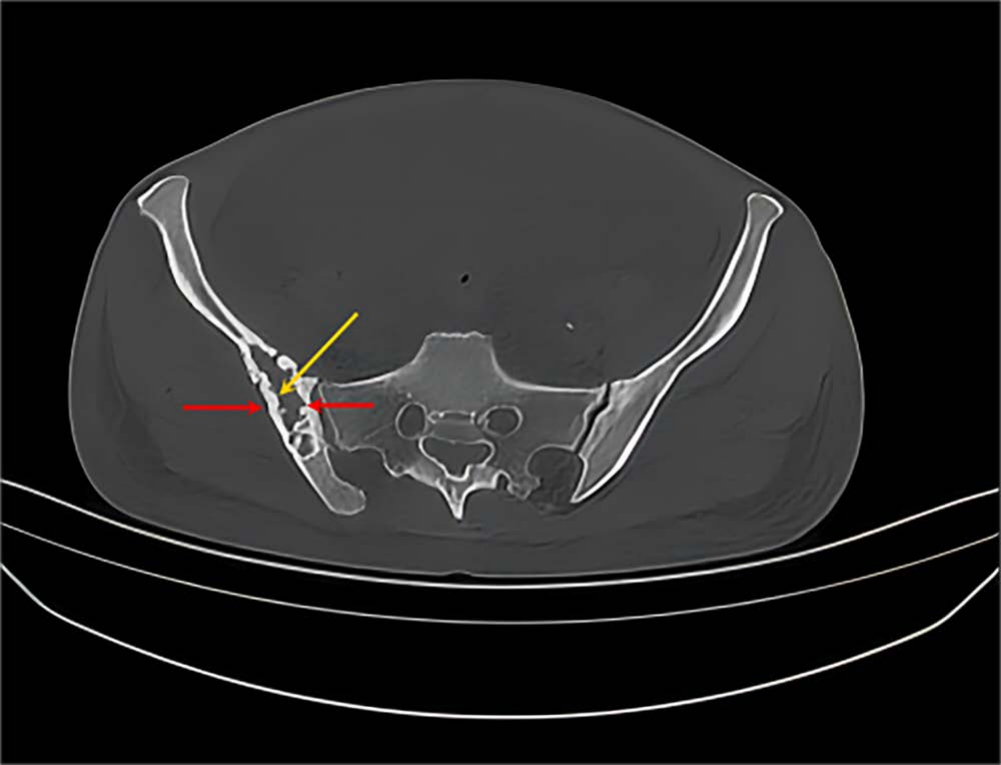
Computed tomography presented a large lytic lesion (yellow arrow), which was surrounded by the sclerotic rims (red arrows) on the right iliac bone.

**Figure 5.**
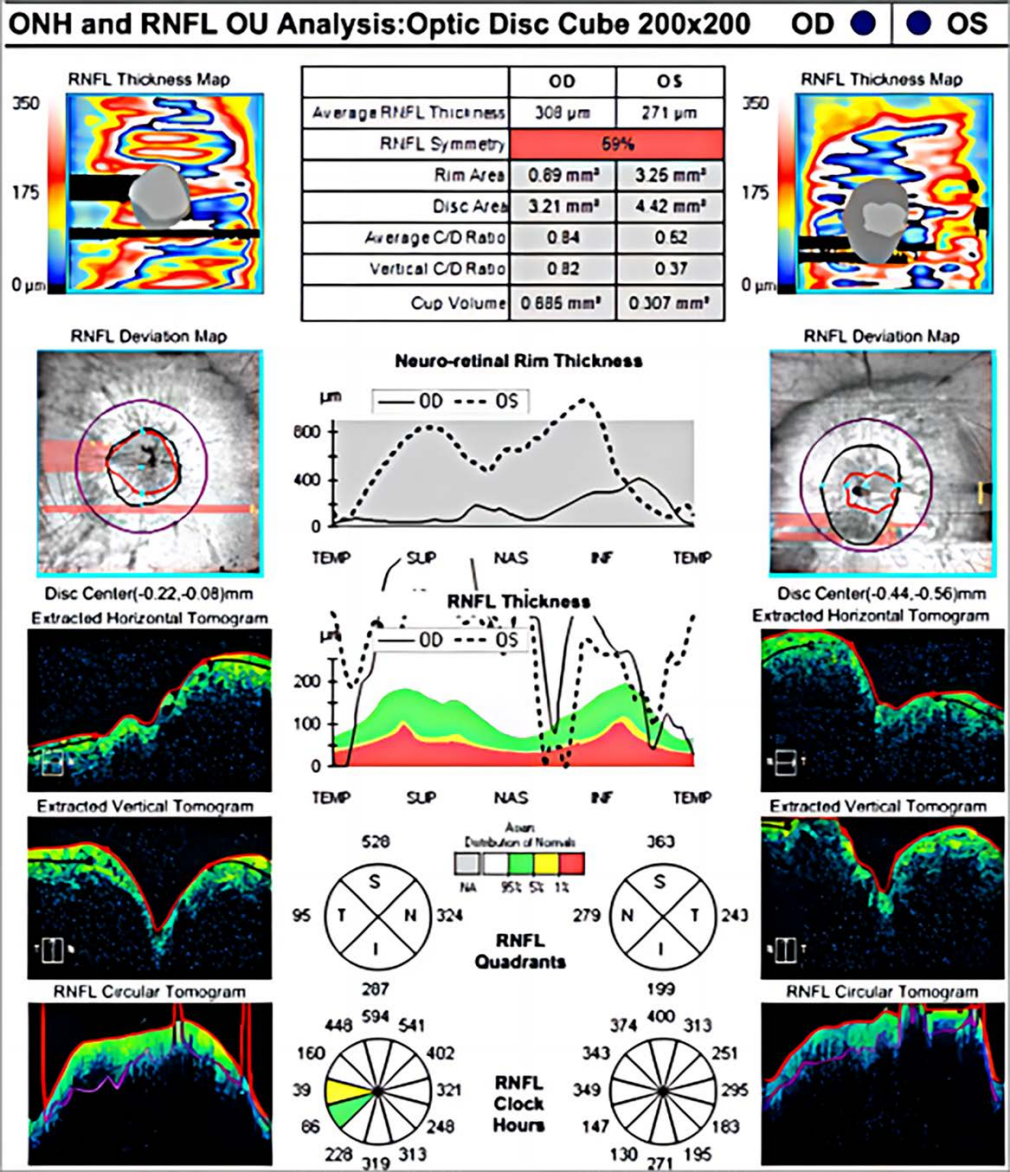
Optical coherence tomography examination of the optic nerve head confirmed papilledema. The average retinal nerve fiber layer (RNFL) thickness of both eyes had increased significantly.

A 45-year-old woman was admitted to the hematology department of Suining Central Hospital in November 2021 due to “lower extremity edema for 3 years and shortness of breath, abdominal distension, and numbness of limbs for 1 year.” The patient had symmetrical depressed edema of both lower extremities, accompanied by edema of both eyelids in June 2018. In May 2020, the patient was referred to the nephrology department. A blood routine examination showed normal white blood cells and hemoglobin and elevated platelet count (398 × 10^9^/L). Liver function and kidney function were normal; 24-hour urine protein was 0.54 g/24 hour. She was diagnosed with chronic glomerulonephritis, and ramipril was administrated. On June 17, 2021, the symptoms of dyspnea and abdominal distension were aggravated, accompanied by enlarged cervical and axillary lymph nodes. Then, the patient was hospitalized in the department of cardiology. Computed tomography (CT) suggested bilateral pleural effusion, pericardial effusion, and ascites. Neck and axillary lymph nodes were punctured, suggesting proliferative lesions without definite neoplastic lesions. In September 2021, the patient’s abdominal distension was worsened, accompanied by numbness and weakness of both lower limbs, inability to walk independently, and loss of vision in both eyes, and she was hospitalized again in the department of nephrology of the hospital on September 24, 2021. CT scan indicated enlarged lymph nodes at the neck, axilla, abdominopelvic cavity, retroperitoneum, and inguinal. Then, a left axillary lymph node excision biopsy was performed and sent to West China Hospital of Sichuan University for pathology consultation. (Left axillary lymph node) Lymphoid tissue proliferative lesions, the structure still existed, part of the follicular growth center atrophy with small blood vessels growing in and some plasma cell infiltration. Immunohistochemistry: lymphocytes CD20 (+, partially), CD3 (+, partially), CD5 (+, partially), CD23 (−), CD10 (+, germinal centers), BCL-2 (−, germinal centers), CyclinD1 (−), CD30 (+, few), IgD (+, partially), plasma cells CD138 (+), CD38 (+), kappa (+), and lambda (+); without light chain restriction expression, IgG4 (+, 5-10/HPF), CD21, and CD23 showed the presence of follicular FDC, ki-67 (+; 3%–5%), in situ hybridization EBER1/2 (−). Clonal amplification peaks of IgH and IgK were not detected. Opinion: consider a benign lymphoid tissue proliferative lesion with Castleman disease morphologic changes in some areas (Fig. 1). Liver and kidney function showed total protein of 76.9 g/L, albumin of 34.8 g/L, urea of 18.13 mmol/L, creatinine of 150 µmol/L, and lactate dehydrogenase of 98 U/L. Thyroglobulin test was present decreased free triiodothyronine and free thyroxin and increased thyrotropin. The 24-hour urine protein was normal. Antinuclear antibodies and antineutrophil cytoplasmic autoantibodies were negative. Bone marrow cytomorphology indicated a proliferative bone marrow picture. Bone marrow flow cytometry showed 0.30% polyclonal plasma cells. Bone marrow biopsy slightly increased plasma cells (5%), immunohistochemistry CD38 (+), CD138 (+), kappa (+), and lambda (+). Serum immunofixation electrophoresis (Fig. 2) and urine immunofixation electrophoresis (Fig. 3) were negative. Serum-free light chain kappa was 146.9 mg/L, and lambda was 148.39 mg/L. Serum IgG4 was 706 mg/L (normal). Epstein-Barr virus DNA <4.00 + 02E copies/mL. Peripheral blood interleukin 6 was 13.2 mg/L (reference value ≤5.9 mg/L). Human herpesvirus type 8 was negative. Peripheral blood vascular endothelial growth factor (VEGF) was 975.61 pg/mL (reference value, 0–142 pg/mL). Cardiac ultrasound suggested slight thickening of the left ventricular wall, mild-moderate regurgitation of the pulmonary and tricuspid valves, mild regurgitation of the aortic and mitral valves, pulmonary hypertension (mild), reduced left ventricular diastolic function, and pericardial effusion (small amount) with an ejection fraction of 58%. CT scan suggested an abdominopelvic effusion, bone destruction, and osteosclerosis of the right ilium, measuring ≈5.1 cm × 2.0 cm × 3.5 cm (Fig. 4). Optical coherence tomography suggested bilateral optic papillary edema (Fig. 5). Electromyography suggested that bilateral median, ulnar, radial, and common peroneal nerves exhibited demyelinating changes and axonal damage, and bilateral tibial nerve F wave was disappeared. The diagnosis of M protein-negative POEMS syndrome was made, and the patient was given lenalidomide plus dexamethasone (RD) regimen, along with the eugenol hormone. After 3 courses of RD regimen, the patient’s numbness in the limb was relieved, and she was able to walk independently. In March 2022, serum VEGF level was found to be 331.74 pg/mL, and her peritoneal, pericardial, and pleural effusions disappeared. After 9 courses of the RD regimen, the patient’s serum VEGF level was found to be 140.11 pg/mL, and she is currently on lenalidomide monotherapy, with her serum VEGF level remaining in the normal range and a good quality of life.

## 3. Discussion

POEMS syndrome is a clonal plasma cell tumor involving multiple systems.^[2]^ Due to its rare onset, its clinical manifestations are diverse and variable. The most common symptoms are numbness and weakness of both lower extremities, edema, and ascites; therefore, patients are often first diagnosed in neurology, nephrology, and gastroenterology. Therefore, the clinical misdiagnosis and delayed diagnosis rates of POEMS syndrome are high, and the delayed diagnosis time can be up to 2 years.^[3]^ The pathogenesis of POEMS syndrome is not completely clear, but it has been hypothesized that abnormally high serum concentrations of VEGF trigger a variety of symptoms.^[4]^ Some studies have confirmed that platelets^[5]^ or plasma cells^[6,7]^ are the main source of VEGF; therefore, the authors believe that clonal plasma cells drive the development of the whole disease. Compared to classical POEMS syndrome, M protein-negative POEMS syndrome has rarely been reported, and its incidence is unknown. Given the scarcity of the disease, it is more prone to misdiagnosis and delayed diagnosis than the M protein-positive POEMS syndrome. The diagnosis of POEMS syndrome must meet 2 mandatory criteria, at least 1 major criterion, and at least 1 minor criterion.^[8]^ Here, we report a case exhibiting multiple peripheral neuropathy (mandatory criterion), Castleman disease (major criterion), sclerosing osteopathy (major criterion), elevated serum VEGF (major criterion), enlarged lymph nodes (minor criterion), edema (minor criterion), hypothyroidism (minor criterion), and thrombocytosis (minor criterion). The patient did not meet the 1 mandatory criterion for monoclonal plasma cell-proliferative disease, which does not appear to be consistent with the diagnosis of POEMS syndrome. Yann et al^[9]^ reported 2 M protein-negative POEMS syndrome cases, arguing that multicentric Castleman disease and Sjögren’s disease are equivalent to monoclonal plasma cell proliferation in the diagnostic criteria for POEMS syndrome. In addition, a large number of cases have been reported in which 15% to 26% of serum immunofixation electrophoresis was negative, even in patients with typical features of POEMS syndrome, and some other patients showed polyclonal increases in immunoglobulins. If the diagnosis of POEMS syndrome depends on the detection of M proteins by electrophoresis, up to 25% of the cases reported in the literature are excluded. Considering the shortened life expectancy of these patients, delays in diagnosis and treatment may lead to a poor prognosis.^[1,10,11]^ He et al^[12]^ reported 13 patients named POEMS syndrome variant without M protein, some of whom met the major and all of whom met the minor criteria for POEMS syndrome, except for undetectable M protein. Hara et al^[13]^ reported a patient who was M-protein-negative but met the other diagnostic criteria for POEMS syndrome. A biopsy was performed on sclerotic bone, which finally showed monoclonal plasma cell proliferation, followed by disease progression, and serum M proteins soon turned positive.^[13]^ From the aforementioned, it is inferred that the development of M protein-negative POEMS syndrome may be due to low or no secretion of M protein by extramedullary monoclonal plasma cells, and therefore, the pathogenesis is not different from the classical POEMS syndrome. Therefore, when the diagnosis of POEMS is highly suspected, evidence of monoclonal plasma cells should be actively investigated to avoid missed diagnoses. Unfortunately, aspiration biopsy of the sclerosing bone disease was not performed in this patient.

Several papers reported the clinical features of POEMS syndrome, including bone damage (predominantly osteosclerosis), monoclonal plasma cells found in the bone marrow, polyneuropathy, Castleman disease, extravascular water overload, endocrine disorders, organomegaly, etc,^[1,11,14]^ and these manifestations and alterations have been written into the diagnostic criteria by Dispenzieri.^[8]^ Comparing the 13 POEMS syndrome variants reported by He et al^[12]^ with classical POEMS syndrome, no clinical manifestations were found between the 2 groups. Nevertheless, whether there was a statistical difference between the 2 groups requires further investigation.

It should be noted that although serum VEGF is a good indicator for the evaluation of disease activity and response to treatment,^[15,16]^ the VEGF measurement value varies greatly among laboratories because there is no standardized standard for the measurement of serum VEGF. Several studies have demonstrated that a VEGF level of >1200 ng/mL presents a high sensitivity and specificity for the diagnosis of POEMS syndrome.^[17,18]^ In contrast, serum VEGF levels in patients with paraprotein-negative POEMS syndrome were not significantly different from those in patients with patients with classical POEMS syndrome.^[12]^ Further studies are needed to determine whether the requirement for VEGF levels at diagnosis is consistent with that of M-positive POEMS syndrome. The M protein in patients with POEMS variant is not always immeasurable, and with time, it may be transformed into M protein-positive POEMS syndrome. He et al^[12]^ followed up on 13 cases of M protein-negative POEMS syndrome, and 2 were positive during the course of the disease, but they did not find unfavorable outcomes for the patients. Therefore, it is necessary for patients with M protein-negative POEMS to undergo dynamic monitoring of blood and urine immunoelectrophoresis.

The aim of treatment for patients with POEMS syndrome is to use antiplasma cells to prolong the survival of patients, increase the depth of remission, and improve related symptoms, thus improving the quality of life of patients. Existing treatments often target plasma cells, and the regimens mostly originate from multiple myeloma. For patients with limited-stage disease (only simple skeletal lesions), plasma cells are highly sensitive to radiotherapy although the radiotherapy dose has not been standardized.^[19]^ A retrospective analysis compared 347 patients with POEMS syndrome who received chemotherapy with melphalan plus dexamethasone, autologous stem cell transplantation (ASCT), and RD regimens, respectively. Indeed, the ASCT group experienced the highest response rate, followed by the RD regimen and, finally, the melphalan plus dexamethasone regimen. However, patients treated with the RD regimen had the shortest progression-free survival and lower overall survival.^[20]^ In view of the generally lower tumor burden in patients with POEMS syndrome, chemotherapy is not routinely used before ASCT, but it can be used to improve the general condition of some patients who are initially unsuitable for transplantation.^[21]^ For patients who are not suitable for transplantation, alternative drugs include immunomodulatory alkylating agents, such as melphalan,^[22]^ lenalidomide,^[23]^ and the proteasome inhibitor bortezomib.^[24]^ As for the CD38 monoclonal antibody daratumumab, there are fewer data on patients with POEMS syndrome. Three patients administered daratumumab in combination with bortezomib and dexamethasone prior to ASCT showed a clinical response and a decrease in VEGF compared to the previous patient.^[25]^ Thirteen cases of M protein-negative POEMS syndrome have been reported in the literature: 12 patients were treated with ASCT, melphalan-based regimens, and lenalidomide/thalidomide-containing regimens, all of which achieved clinical improvement.^[12]^ In our case, complete normalization of serum VEGF levels was achieved after antiplasma cell therapy. M-protein-negative POEMS syndrome is still a plasma cell disease; therefore, antiplasma cell therapy remains the treatment of choice.

In summary, paraprotein-negative POEMS syndrome, a rare clonal plasma cell tumor with diverse clinical manifestations, requires clinicians to strengthen their knowledge of the disease to achieve early detection, diagnosis, and treatment. Diagnosis should be made with caution, searching for as many diagnostic criteria as possible (e.g., Castleman disease, sclerosing osteopathy, elevated serum VEGF, and cutaneous hemangioma). A biopsy of the sclerosing bone should be performed whenever possible. Treatment includes general supportive therapy (e.g., hormone replacement therapy and neurotrophic restorative therapy) and antiplasma cell therapy, and active treatment can significantly improve the quality of life of patients.

## Author contributions

**Conceptualization:** Chao Ding.

**Data curation:** Chao Ding.

**Formal analysis:** Chao Ding.

**Investigation:** Chao Ding.

**Writing – original draft:** Chao Ding.

**Software:** Yanqiu Li.

**Supervision:** Yanqiu Li.

**Validation:** Yanqiu Li.

**Writing – review & editing:** Yanqiu Li.
